# Reducing Surgical Site Infections in Neurosurgery During the Intra-operative Phase: An Audit of National Institute for Health and Care Excellence (NICE) Guideline Adherence at a Major Trauma Centre

**DOI:** 10.7759/cureus.73605

**Published:** 2024-11-13

**Authors:** Hamza Ahmed, K Joshi George, Omar Walid Rasheed, Aima Gilani, Mahmoud Mohammad Mustafa Aldameiry, Mohsin Fayyaz, Aarish Azeem

**Affiliations:** 1 Trauma and Orthopaedics, Salford Royal National Health Service (NHS) Foundation Trust, Manchester, GBR; 2 Neurosurgery, University of Manchester, Manchester, GBR; 3 Neurosurgery, Salford Royal National Health Service (NHS) Foundation Trust, Manchester, GBR; 4 Stroke Medicine, Salford Royal National Health Service (NHS) Foundation Trust, Manchester, GBR

**Keywords:** cost effectiveness, neurosurgery, nice guidelines, patient safety, ssis, surgical site infections

## Abstract

Surgical site infections (SSIs) pose significant risks to patient safety and healthcare systems globally despite preventive measures. This study audits compliance with the National Institute for Health and Care Excellence (NICE) guidelines for SSI prevention during the intra-operative phase in the Neurosurgery Department at Salford Royal National Health Service (NHS) Foundation Trust. Conducted in two audit cycles from October 2022 to October 2023, the study assessed adherence to 19 key parameters aimed at minimising SSIs. Significant improvements were observed in various areas, including antibiotic prophylaxis information (from 44% to 90%) and bodywash compliance (from 14% to 80%). Compliance with skin preparation protocols also showed marked progress, with antiseptic application time improving from 12% to 80%. However, areas such as double gloving (increased from 36% to 44%) and the use of antimicrobial sutures (from 2% to 30%) indicate opportunities for further enhancement. The findings underscore the effectiveness of targeted interventions and the importance of continuous education and evaluation of surgical practices to reduce SSIs, ultimately improving patient outcomes and alleviating financial burdens on the NHS. Future audits are recommended to ensure sustained compliance and quality of care in Neurosurgery.

## Introduction

Each year, numerous surgical procedures are performed globally. Despite rigorous efforts to prevent complications before, during, and after surgery, there remains a risk of adverse outcomes. Among these, surgical site infections (SSIs) are among the most dangerous [1]. They can be regarded as healthcare-associated infections that occur following invasive procedures. Sources of contamination may include the patient's own skin flora entering the surgical wound [2] or environmental sources such as operating staff, the theatre, or broader hospital and home environments.

In recent years, SSIs have become relatively common. One specific study characterised SSIs after open surgery [3] in the National Health Service (NHS) in 2023 and concluded that 11% of patients developed SSIs, of which 15% were inpatients and 85% were in the community after hospital discharge. This issue poses a serious threat, leading to considerable morbidity and mortality among surgical patients. Therefore, it is essential to address this risk promptly through preventive measures taken before, during, and after surgery.

Aim and objectives

This study audits compliance with the National Institute for Health and Care Excellence (NICE) guidelines for SSI prevention (NG125, published on April 11, 2019) during the intra-operative phase [4] at the Department of Neurosurgery in Salford Royal NHS Foundation Trust, Manchester. The primary objectives were to reduce the risk of SSIs, shorten extended hospital stays [5], and lessen the financial impact on the NHS. Additionally, this assessment measured the adherence to NICE guidelines within the trust and identified specific areas within the department that could benefit from further improvement.

## Materials and methods

In addition to established practices, such as the use of antibiotic prophylaxis, avoidance of hypothermia, and peri-operative blood glucose control for reducing SSIs, recent research and outcomes merit consideration for inclusion of other important parameters. By assessing adherence to key protocols like hand hygiene, skin preparation, and use of sterile equipment, this research aims to improve surgical outcomes by minimising SSIs.

The study conducted two audit cycles to measure baseline compliance (cycle 1) and evaluate improvements (cycle 2) following interventions. The approval for the audit and re-audit was obtained from the local committee in October 2022 (approval numbers 2022 186 and 2023 159). Data collection was conducted prospectively using a checklist assessing 19 specific parameters (Table 1).

**Table 1:** Checklist consisting of 19 parameters assessed as per NICE guidelines NICE, National Institute for Health and Care Excellence; NHS, National Health Service; SSIs, surgical site infections

Checklist on prevention of surgical site infections during the intraoperative phase against NICE guidelines: Neurosurgery unit at Salford Royal NHS Foundation Trust
Has anyone informed the patient before the operation that they will need antibiotic prophylaxis/will need antibiotics during the operation?
Has it been confirmed in theatre that the patient has had a bodywash prior to surgery?
If yes, what was the bodywash done with? Answer with one of the following: Soap, 4.0% aqueous chlorhexidine (Hibiscrub (Mölnlycke Health Care, Göteborg, Sweden)), you do not know.
Have all members of the operating team washed their hands with an aqueous antiseptic surgical solution or alcoholic hand rub?
Have all members of the operating team used a brush or pick for the nails to ensure they are visibly clean?
Has patient’s iodine allergy status been reviewed before the incise drape is put on by the surgeon and operating team?
Which type of incise drape is used? (non-iodophor-impregnated or iodophor-impregnated drape)
Is the operating team wearing sterile gowns?
Are two pairs of sterile gloves worn by all members of operating team or just one?
Has an antiseptic skin preparation agent been used right before the incision is made?
Is 2.0% chlorhexidine in 70% alcohol applicators (ChloraPrep (CareFusion, San Diego, CA)) used for antiseptic skin preparation?
Is alcohol-based solution of povidone-iodine used for antiseptic skin preparation?
Was allergy to chlorhexidine confirmed before antiseptic skin preparation?
How long did you rub the incision site with antiseptic preparation? If you do not remember the time, please answer with you do not know.
What was the direction of cleansing of the antiseptic preparation? Towards the wound site or away from the wound site?
Was the antiseptic solution allowed to dry before drapes were fixed?
Was the skin antiseptic solution removed from the surgical field immediately before the surgery began? Please answer as yes, no, or you do not know.
Is wound irrigation done to reduce the risk of SSIs?
Were antimicrobial triclosan-coated sutures used to close the wound?

A prospective closed-loop audit was initiated in October 2022, with the first audit cycle concluding in February 2023. Data collection and analysis were conducted using SPSS Version 28.0.0.1 (IBM SPSS Statistics for Windows, IBM Corp., Armonk, NY), with results presented in pie charts and bar graphs to the local committee in April 2023. Following the meeting, potential improvements were discussed and subsequently implemented. These included a targeted intervention from February 2023 to June 2023 involving the distribution of pamphlets, poster presentations, and both informal and formal training sessions for theatre staff.

The second cycle of data for the re-audit was collected from June 2023 to October 2023, with findings presented to the local committee at the end of October 2023. In both audit cycles, all inpatients were included, with no direct patient involvement, and all data were maintained confidentially. Each cycle comprised a sample of 50 patients.

## Results

Key findings and analysis

Table 2 summarises the results of both audit cycles.

**Table 2:** Summary of the results of both cycles, showing the percentage change where possible

NICE guidelines [4]	Cycle 1 - n (%)	Cycle 2 - n (%)	% Change
Has anyone informed the patient before the operation that they will need antibiotic prophylaxis/will need antibiotics during the operation?	22 (44%) answered yes	45 (90%) answered yes	+46%
Has it been confirmed in theatre that the patient has had a bodywash prior to surgery?	7 (14%) answered yes	40 (80%) answered yes	+66%
If yes, what was the bodywash done with? Answer with one of the following: Soap, 4.0% aqueous chlorhexidine (Hibiscrub (Mölnlycke Health Care, Göteborg, Sweden)), you do not know.	4 (8%) with soap/Hibiscrub	40 (80%) with soap/Hibiscrub	+72%
Have all members of the operating team washed their hands with an aqueous antiseptic surgical solution or alcoholic hand rub?	50 (100%)	50 (100%)	0%
Have all members of the operating team used a brush or pick for the nails to ensure they are visibly clean?	41 (82%)	45 (90%)	+8%
Has patient’s iodine allergy status been reviewed before the incise drape is put on by the surgeon and operating team?	45 (90%)	48 (96%)	+6%
Which type of incise drape is used? (non-iodophor-impregnated or iodophor-impregnated drape)	20 (40%) used iodophore impregnated drapes	35 (70%) used iodophore impregnated drapes	+30%
Is the operating team wearing sterile gowns?	50 (100%)	50 (100%)	0%
Are two pairs of sterile gloves worn by all members of operating team?	18 (36%)	22 (44%)	+8%
Has an antiseptic skin preparation agent been used right before the incision is made?	45 (90%)	49 (98%)	+8%
Is 2.0% chlorhexidine in 70% alcohol applicators (ChloraPrep (CareFusion, San Diego, CA)) used for antiseptic skin preparation or alcohol-based solution of povidone-iodine?	41 (82%) ChloraPrep	42 (84%) ChloraPrep	+2%
Was allergy to chlorhexidine confirmed before antiseptic skin preparation?	46 (92%)	48 (96%)	+4%
How long did you rub the incision site with antiseptic preparation? If you do not remember the time, please answer with you do not know.	6 (12%) for two to three minutes	40 (80%) for two to three minutes	+68%
What was the direction of cleansing of the antiseptic preparation? Towards the wound site or away from the wound site?	43 (86%) away from the wound site	48 (96%) away from the wound site	+10%
Was the antiseptic solution allowed to dry before drapes were fixed?	26 (52%)	45 (90%)	+38%
Was the skin antiseptic solution removed from the surgical field immediately before the surgery began? Please answer as yes, no, or you do not know.	50 (100%)	50 (100%)	0%
Was the antiseptic solution allowed to dry before drapes were fixed?	26 (52%)	45 (90%)	+38%
Is wound irrigation done to reduce the risk of SSIs?	50 (100%)	50 (100%)	0%
Were antimicrobial triclosan-coated sutures used to close the wound?	1 (2%)	15 (30%)	+28%

Antibiotic Prophylaxis Information

The percentage of patients informed about the need for antibiotic prophylaxis increased significantly from 44% to 90% (+46%). This improvement indicates heightened awareness and emphasis on patient education.

Bodywash Compliance

Confirmation that patients received a bodywash before surgery rose from 14% to 80% (+66%), suggesting improved adherence to preoperative hygiene protocols. However, the specific wash used (soap or Hibiscrub (Mölnlycke Health Care, Göteborg, Sweden)) was often unknown in cycle 1 (92%) but better documented in cycle 2, with 80% specifying the type.

Hand Hygiene of the Operating Team

Hand washing compliance: The operating team maintained a high standard with 100% compliance in both cycles, showing consistency in critical infection control practices.

Use of Nail Brushes

There was a modest increase in the use of brushes or picks for nail cleaning, from 82% to 90% (+8%). This area shows slight improvement but highlights a need for more comprehensive compliance.

Skin Preparation and Antiseptic Use

Chlorhexidine allergy confirmation: Allergy verification increased from 92% to 96% (+4%), showing minor progress.

Skin Preparation Timing and Technique

Compliance with the recommended antiseptic application time (two to three minutes) increased substantially from 12% to 80% (+68%), while the direction of cleansing (away from the wound) improved from 86% to 96% (+10%). These results indicate a stronger adherence to antiseptic application standards.

Drying of Antiseptic Solution

Allowing the antiseptic to dry before draping improved from 52% to 90% (+38%), reflecting better compliance with drying protocols crucial for reducing SSI risk.

Use of Surgical Gowns, Gloves, and Drapes

Sterile gown compliance: Compliance remained at 100% across both cycles, showcasing adherence to gown protocol.

Double gloving: Double gloving improved from 36% to 44% (+8%), although the relatively low compliance may suggest a need for further education on the benefits of added protection against infection.

Type of incise drapes: Use of iodophor-impregnated drapes rose from 40% to 70% (+30%), suggesting an increased focus on drapes that actively release antiseptics, aligned with NICE recommendations.

Wound Closure and Additional Measures

Use of antimicrobial sutures: The utilisation of triclosan-coated sutures increased from 2% to 30% (+28%), a substantial improvement, though with room for broader adoption.

Wound irrigation: Compliance with wound irrigation remained constant at 100%, reinforcing the consistency of this protocol in the operating procedure.

## Discussion

Although there is no universally accepted diagnostic tool or protocol to confirm the presence of an SSI, the definition provided by the Centres for Disease Control and Prevention (CDC) is widely utilised [6].

A superficial SSI [7] is defined as an infection that occurs within 30 days following surgery, affecting only the skin and subcutaneous tissue of the incision. It is associated with a prompt diagnosis clinically by the surgeon. In addition to this, it can also show certain signs and symptoms such as pain, tenderness, swelling, or a positive blood culture on investigations or tissue analysis under microscopy. Purulent discharge can also be seen grossly. At least one of the aforementioned criteria is necessary to classify it as a superficial SSI. 

A deep incisional SSI is defined as an infection occurring within 30 days post-operation if no implant was placed or within one year if an implant was left in place. This infection must be related to the surgical procedure and involve the deeper soft tissues [7], like the fibrous connective tissue and muscle layers of the incision. In addition to this, there should also be a clinical diagnosis by the surgeon, purulent discharge, evidence of infection, and signs and symptoms such as fever, pain, and swelling. 

Antibiotic prophylaxis

Prophylactic antibiotics reduce the incidence of SSIs, consequently minimising hospital stays and healthcare costs [8]. They help lower both morbidity and mortality rates associated with surgery. When selecting antibiotics for surgical prophylaxis, it is essential to choose agents with the narrowest effective antibacterial spectrum to limit the development of resistance. It is ideal to administer antibiotic prophylaxis prior to clean surgeries involving the insertion of a prosthesis or implant [9], clean-contaminated surgeries, and contaminated surgeries [10].

It is desirable to avoid the routine use of antibiotic prophylaxis for clean, non-prosthetic, uncomplicated surgeries as it has not shown any evidence-based improvement in outcomes. In addition to this, patients should be informed in advance that they may require antibiotic prophylaxis, and they should be notified postoperatively if antibiotics were administered during their procedure.

Bodywash

Preoperative skin cleansing has been shown to reduce infection risk when used before surgical procedures [11]. The skin barrier protection is compromised when broken (e.g., through cuts, abrasions, or punctures), increasing the likelihood of bacterial entry and infection. This is most likely around the incision sites, intravenous access sites, and warm areas like the groin and underarms.

Agents for bodywash

The commonly used product for bodywash is Hibiscrub (chlorhexidine 4% skin cleanser), an antiseptic solution containing chlorhexidine gluconate, methylated spirit, and water. This effectively removes skin microorganisms [12] and provides extended protection against re-colonization. In cases where the patient has a known chlorhexidine allergy, soap can be used as an alternative. The aim of preoperative skin antisepsis is to reduce the risk of SSIs by reducing the number of micro‐organisms on the skin [13].

Hand decontamination and appropriate personal protective equipment (PPE)

It is vital that before the first operation, the surgical team should wash their hands [14] with an aqueous antiseptic solution, using a single-use brush or pick for nails, ensuring that hands and nails are visibly clean. For subsequent operations, hands should be cleansed with an alcohol-based hand rub or an antiseptic solution; if hands are soiled, they should be rewashed with an antiseptic surgical solution. A medical brush is effective in significantly reducing microorganisms on hands and forearms prior to surgery, especially for removing dirt from the skin and under nails during surgical scrubbing. A nail pick may also be used.

There are various coverings used to create a barrier between the environment and the patient’s wound, ensuring a sterile field. Masks, for example, are designed to capture expelled water droplets and contain one or two finely woven filters to block bacteria. They cover the nose and mouth, but there are concerns that improper use could lead to air leaks along the mask's sides. Shoe coverings are intended to reduce the transfer of external materials in and out of the operating room. Gowns are worn over standard surgical attire, allowing for removal and replacement when contaminated.

Iodine allergy status

It is crucial to ascertain the patient’s iodine allergy status. Symptoms indicative of an iodine allergy include tongue and throat swelling, fever, tachycardia, difficulty in breathing, facial flushing, and, in severe cases, loss of consciousness.

Incise drapes 

Drapes serve as a protective barrier between the incision and the patient's skin, which, despite being cleansed, may still contain microorganisms at deeper skin levels beyond the reach of standard cleansing [15]. The NICE guidelines [4] advise against the routine use of non-iodophor-impregnated incise drapes in surgery, as they may elevate the risk of SSIs. If an incise drape is deemed necessary, an iodophor-impregnated drape should be used.

Sterile gloves

The NICE guidelines [4] recommend considering the use of double-layer sterile gloves in situations with a high risk of glove perforation, particularly where contamination could have serious consequences. Double gloving significantly reduces the infection risk for operating room personnel [16]. The inner glove provides a critical layer of protection against blood-borne pathogens in the event of an outer glove puncture.

The use of skin sealants and skin preparation agents

It is suggested that applying a skin sealant prior to surgery helps to prevent any remaining microorganisms from entering the surgical wound after the skin has been decontaminated [17].

The first choice, unless contraindicated for skin preparation, is an alcohol-based solution of chlorhexidine [18]. If the surgical site is close to a mucus membrane, then aqueous solution of chlorhexidine can be used as well. An alcohol-based solution of povidone is used if chlorhexidine is contraindicated. 

Rubbing, cleansing direction, and drying

The incision site should be rubbed with an antiseptic solution for a minimum duration of two to three minutes as per NICE guidelines [4]. When multiple swabs are used, the cumulative time spent cleaning should also be two to three minutes.

The antiseptic solution should be applied in a manner that directs away from the wound site to prevent contamination [19]. Ideally, allow the antiseptic solution to dry for two to three minutes before proceeding.

Triclosan-coated sutures

Triclosan-coated sutures are recommended by three major health organisations - the CDC, WHO, and the American College of Surgeons (ACS) - to prevent SSIs. This recommendation is not limited to any specific brand.

Sutures may contribute to the risk of infection since implanted materials can lower the infection threshold. For instance, the infective dose in typical patients is usually between two to eight million microorganisms per gram of tissue, but sutures can significantly reduce this threshold. Evidence from laboratory research indicates that antimicrobial-coated, synthetic, absorbable sutures can effectively deliver triclosan, an antiseptic, into tissue.

Three independent systematic reviews and meta-analyses have validated the clinical efficacy of these sutures with level 1A evidence. Wang et al. in 2013 [20] identified 17 RCTs involving 3,720 patients, showing a 30% reduction in SSIs. Edmiston et al., in the same year [21], analysed 13 higher-quality RCTs involving 3,568 patients, showing a 27% reduction in SSIs. Similarly, Daoud et al. the following year [22] used PRISMA guidelines to analyse 15 RCTs with 4,800 patients, finding a 33% reduction in SSIs. This meta-analysis showed no publication bias and demonstrated robust sensitivity.

Additional peer-reviewed, double-blind RCTs and systemic reviews [23,24] confirmed the success of triclosan-coated sutures, ascertaining their role in reducing SSIs across clean, clean-contaminated, and contaminated surgeries. These sutures have shown effectiveness against pathogens, including *Staphylococcus aureus*, *Staphylococcus epidermidis*, *Escherichia coli*, *Klebsiella pneumoniae*, and methicillin-resistant *Staphylococcus aureus*.

Recommendations

At the end of our study, we made the abovementioned recommendations, which have since been followed by the department. It was stressed that a reaudit should be conducted again in one year’s time to assess compliance. In addition to this, the trust collaborated with organisations like Ethicon to provide education to junior staff and trainees on the advantages of triclosan sutures in preventing SSIs.

## Conclusions

In conclusion, this study of adherence to NICE guidelines for preventing SSIs during the intraoperative phase at Salford Royal NHS Foundation Trust demonstrates significant progress in several key areas. Notably, improvements in antibiotic prophylaxis information, bodywash compliance, and adherence to skin preparation protocols underscore the commitment to enhancing patient care and safety. The remarkable increase in various essential domains illustrates the effectiveness of targeted interventions. However, certain areas, including double gloving and the use of antimicrobial sutures, reveal opportunities for further enhancement. The modest gains in these domains suggest a need for continued education and reinforcement of best practices among the surgical team.

Overall, the findings of this audit not only highlight the importance of adherence to established guidelines but also emphasise the necessity for ongoing evaluation and improvement in surgical protocols. By addressing the identified gaps and fostering a culture of compliance, we can significantly reduce the incidence of SSIs, leading to shorter hospital stays and improved outcomes for patients, thereby alleviating some of the financial burdens on the NHS. Future audits should continue to monitor these metrics to ensure sustained improvement and ultimately enhance the quality of care in Neurosurgery.
